# Recurrent *DNMT3A* R882 Mutations in Chinese Patients with Acute Myeloid Leukemia and Myelodysplastic Syndrome

**DOI:** 10.1371/journal.pone.0026906

**Published:** 2011-10-31

**Authors:** Jiang Lin, Dong-ming Yao, Jun Qian, Qin Chen, Wei Qian, Yun Li, Jing Yang, Cui-zhu Wang, Hai-yan Chai, Zhen Qian, Gao-fei Xiao, Wen-rong Xu

**Affiliations:** 1 Department of Haematology, Affiliated People's Hospital of Jiangsu University, Zhenjiang, Jiangsu, People's Republic of China; 2 Laboratory Center, Affiliated People's Hospital of Jiangsu University, Zhenjiang, Jiangsu, People's Republic of China; 3 Key Institute of Clinical Laboratory Science, School of Medical Science and Laboratory Medicine, Jiangsu University, Zhenjiang, Jiangsu, People's Republic of China; The University of Birmingham, United Kingdom

## Abstract

Somatic mutations of *DNMT3A* gene have recently been reported in acute myeloid leukemia (AML) and myelodysplastic syndrome (MDS). We examined the entire coding sequences of *DNMT3A* gene by high-resolution melting analysis and sequencing in Chinese patients with myeloid malignancies. R882 mutations were found in 12/182 AML and in 4/51 MDS, but not in either 79 chronic myeloid leukemia (CML), or 57 myeloproliferative neoplasms (MPNs), or 4 chronic monomyelocytic leukemia. No other *DNMT3A* mutations were detected in all patients. R882 mutations were associated with old age and more frequently present in monoblastic leukemia (M4 and M5, 7/52) compared to other subtypes (5/130). Furthermore, 14/16 (86.6%) R882 mutations were observed in patients with normal karyotypes. The overall survival of mutated MDS patients was shorter than those without mutation (median 9 and 25 months, respectively). We conclude that *DNMT3A* R882 mutations are recurrent molecular aberrations in AML and MDS, and may be an adverse prognostic event in MDS.

## Introduction

Tumorigenesis is known to be a multistep process, which is the result of not only genetic alterations but also epigenetic changes [1]. DNA methylation is a major form of epigenetic modification and plays an essential role in development, differentiation, genomic stability, X-inactivation, and imprinting by specific regulation of gene expression. DNA methylation occurs by covalent addition of a methyl group at the 5′ carbon of the cytosine ring. The transfer of methyl groups from S-adenosyl-l-methionine to cytosine is a heritable process that is catalyzed by several DNA methyltransferases (DNMTs) during cell replication. There are at least three different DNMTs involved in cellular DNA methylation: DNMT1, DNMT3A, and DNMT3B. It has been documented that abnormal methylation is involved in the development of many tumors [1]. All three DNMTs are constitutively expressed at different levels in most human tissues. Overexpression of DNMTs and its association with alterated DNA methylation have been observed in tumors including leukemias [2]. Recently, somatic mutations in *DNMT3A* gene have been found in acute myeloid leukaemia (AML) with a frequency of 22.1% [3]. Recurrent *DNMT3A* muations have also been identified in myelodysplastic syndrome (MDS) [4]. The “hotspots” mutation occurs at the amino acid position 882, resulting in the replacement of arginine by histidine (R882H), cysteine (R882C), serine (R882S), or phenylalanine (R882P). This work studied the occurrence of *DNMT3A* mutations in Chinese AML and MDS patients.

## Materials and Methods

### Patients' samples and cell lines

This study was approved by the Scientific Committee of Jiangsu Province Health Department. Bone marrow aspirates or peripheral blood samples of patients with various hematologic malignancies were collected after informed consent given. The patients included 182 AML, 51 MDS, 79 chronic myeloid leukemia (CML) (60 at chronic phase, 4 at accelerated phase, 15 at blast crisis), 57 myeloproliferative neoplasms (MPNs) (22 polycythemia vera, 28 essential thrombocythemia, and 7 primary myelofibrosis) and 4 chronic monomyelocytic leukemia (CMML). These hematological malignancies were diagnosed according to the French-American-British Cooperative Group Criteria and the 2008 World Health Organization proposal [5], [6]. Bone marrow specimens obtained at the time of complete hematologic remission from five AML patients with *DNMT3A* mutations at initial diagnosis and peripheral blood from 73 healthy individuals were used as control.

Seven human leukemic cell lines (HL-60, NB4, THP-1, SHI-1, U937, HEL, and K562, all provided by Dr. Suning Chen, Jiangsu Institute of Hematology, Jiangsu, China) were cultured in RPMI 1640 medium containing 10% fetal calf serum. All cell lines were harvested during exponential growth.

### Mutation analysis

For *DNMT3A* mutational analysis, 25 pairs of primers were diesigned to amplify the entire coding sequences of *DNMT3A* (Genbank AF067972.2). DNA fragment spanning the codon R882 was amplified by polymerase chain reaction (PCR) using the following primers: 5′- TTTGGTTTCCCAGTCCACTATAC -3′ (forward), and 5′- CCAGCAGTCTCTGCCTC -3′ (reverse). The size of PCR product was 67-bp. PCR was performed in 25-µL volume in the presence of 1× PCR buffer (Invitrogen, Merelbeke, Belgium), 0.2 mmol/L of each dNTP, 2.5 mmol/L of MgCl_2_, 0.4 µmol/L of both forward and reverse primers, 0.8 µmol/L of oligonucleotide calibrators [7], 1× LCgreen Plus (Idaho Technology Inc. Salt Lake City, Utah), 1 U Taq polymerase (MBI Fermentas, Canada), and 50 ng genomic DNA. PCR reactions were carried out on a 7300 Thermo cycler (Applied Biosystems, Foster City, CA, USA). The temperature cycling protocol consisted of an initial denaturation step at 95°C for 5 minutes, followed by 40 cycles of denaturation at 94°C for 30 seconds, annealing at 59°C for 30 seconds, and an extension at 72°C for 30 seconds. The primer sets and PCR conditions for other DNA fragments of *DNMT3A* exons will be provided on request.

High-resolution melting analysis (HRMA) was performed for PCR products by the LightScanner™ platform (Idaho Technology Inc. Salt Lake City, Utah). Plates were heated in the LightScanner from 55°C up to 95°C with a ramp rate of 0.10°C/s. The melting curve analysis was carried out using the LightScanner software package with CALL-IT® software (Idaho Technology Inc. Salt Lake City, Utah). Melting profiles were calibrated by internal oligonucleotide controls, and then normalized, grouped and displayed as fluorescence-versus-temperature plots or subtractive difference plots (-df/dt vs T).

### DNA sequencing

A separate PCR was carried out to generate a larger amplicon spanning R882 (410 bp). PCR conditions were similar with that for HRMA except for the primers which were: 5′-TCTGGGTGGCACGGTCTT -3′ (forward) and 5′-CCTTGCTTAATGGGTGTA -3′ (reverse). PCR products were directly sequenced on both strands using an ABI 3730 automatic sequencer.

### Statistics

Statistical analysis was performed using the SPSS 13.0 software package (SPSS, Chicago, IL, USA). Pearson Chi-square analysis and Fisher exact test were carried out to compare the difference of categorical variables between patient groups. Mann-Whitney's U-test was carried out to compare the difference of continuous variables between patient groups. Survival was analyzed according to the Kaplan-Meier method. For all analyses, a *P*-value of less than 0.05 (two-tailed) was considered statistically significant.

## Results and Discussion

**Figure 1:**
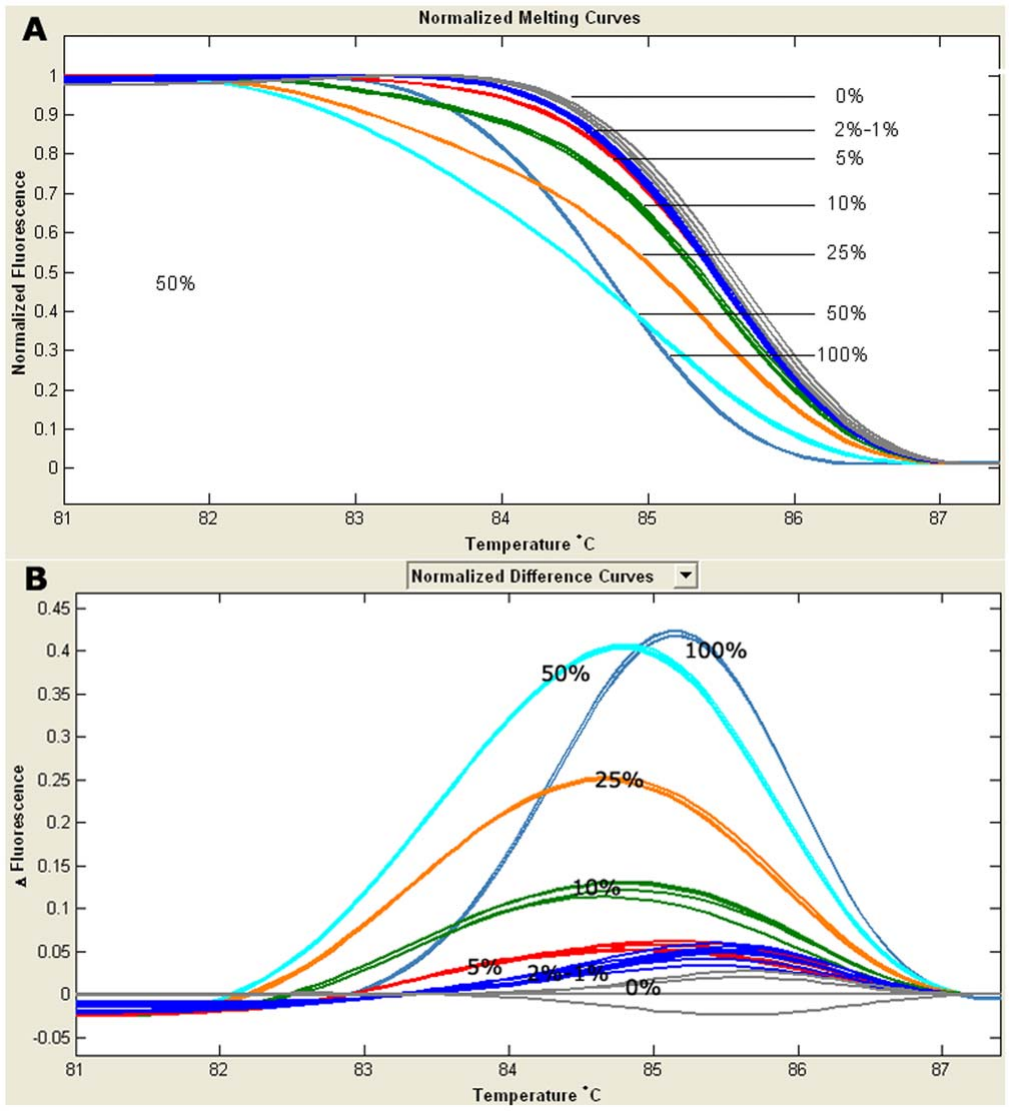
Results of a dilution series of *DNMT3A* R882H mutant in a background of wild-type DNA detected by HRMA. A: normalized melting curves; B: normalized difference curves. HRMA identified mutated R882H with the maximal sensitivity of 2%.

**Figure 2:**
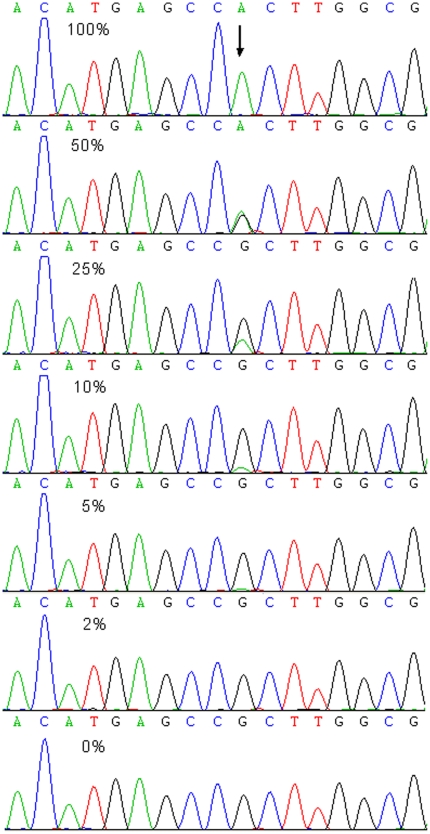
Results of a dilution series of *DNMT3A* R882H mutant in a background of wild-type DNA detected by DNA sequencing. The maximal sensitivity of 10% was obtained. Arrow showed the mutation site.

The sensitivity of HRMA was evaluated by detecting plasmid DNA with different concentrations of R882H mutant diluted by wild type (0%, 1%, 2%, 5%, 10%, 25%, 50%, and 100% mutant). HRMA could easily distinguish R882H mutation with the maximal sensitivity of 2% in a background of wild-type DNA (Figure 1). However, Mutated R882H was identified at the maximal sensitivity of 10% by direct DNA sequencing (Figure 2).

**Figure 3:**
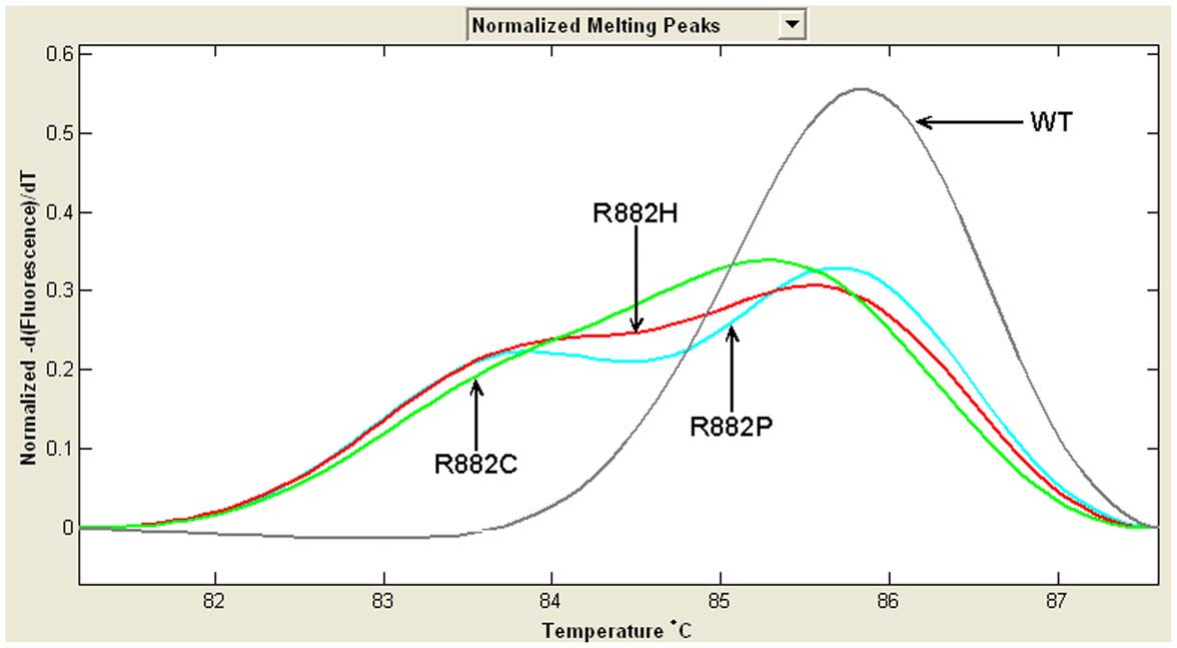
Representative melting shapes of three R882 mutations and wild-type DNA. WT: wild-type. Three R882 mutations could be easily distinguished according to their different melting paths.

**Figure 4:**
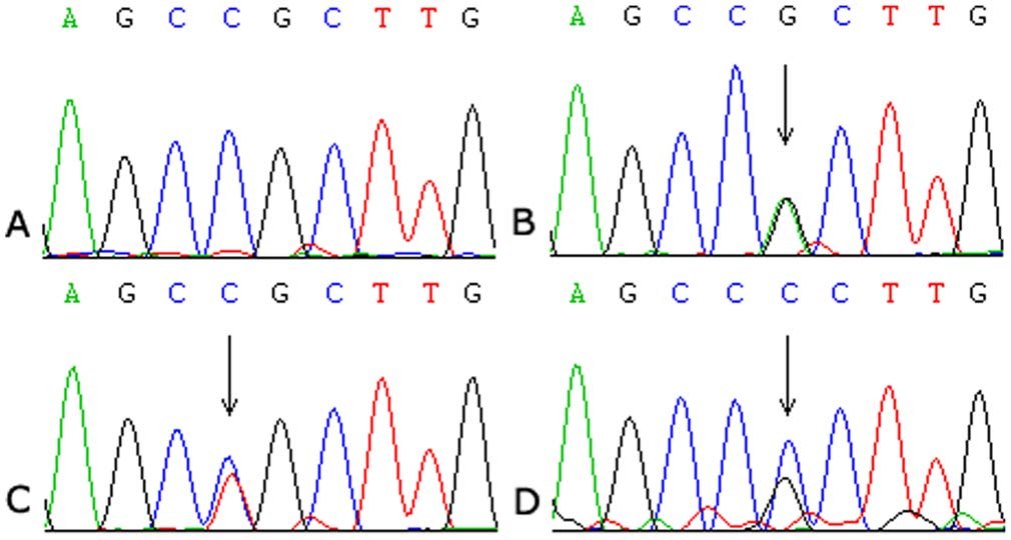
Sequences of the mutated *DNMT3A* R882. A: wild type sequence; B: R882H mutation; C: R882C mutation; D: R882P mutation. Mutations are indicated with black arrows.

In the cohort of 372 patients, 16 cases were discovered to harbor a heterozygous R882 mutation of *DNMT3A*, including R882H (n = 11), R882C (n = 4), and R882P (n = 1). The representative melting curves and sequence chromatograms of three types of R882 mutations were shown on Figure 3 and 4. R882 mutation, positive in the bone marrow samples from 5 AML patients at initial diagnosis, disappeared after complete remission. Furthermore, R882 mutation was not present in the healthy control. No R882 mutations were found in all seven leukemic cell lines.

R882 mutations were identified in 12 (6.6%) of 182 AML patients (Table 1). The basic clinical data of these patients were listed in Table 2. There were three types of mutations, including R882H (n = 8, 66.7%), R882C (n = 3, 25.0%), and R882P (n = 1, 8.3%). No correlation was observed between R882 mutations and gender or white blood cell (WBC) counts. AML patients with *DNMT3A* R882 mutations were more prevalent at older age and present with significantly higher median platelet counts at diagnosis compared to those without mutations (Table 1). Although median WBC counts were higher in AML patients with R882 mutations than those without mutations, no statistical difference was observed. It is noticed that one patient (case 3 in Table 2) had a period of panctyopenia four months before the diagnosis of AML and another patient (case 11 in Table 2) had a period of leukocytopenia fifteen months before the diagnosis of AML. Significant dysplastic changes were identified in bone marrow of these two cases, such as megaloblastoid changes, multinuclear erythroid precursors, and micromegakaryocytes. Thus, AML derived from MDS might be considered in these two patients. R882 mutations were also identified in two MDS-derived AML [8], however, it was not determined whether the mutations were present in bone marrow cells at the MDS stage [8].

**Table 1:** Distribution of *DNMT3A* R882 mutations in AML and MDS.

	R882 mutation	Wild-type	*P*
AML	12	170	
Sex, male/female	10/2	100/70	0.081
Median age at diagnosis, years (range)	60 (49–93)	42 (18–86)	0.002
Median WBC at diagnosis, ×10^9^/L (range)	74.8 (1.3–197.7)	15.9 (0.5–528)	0.166
Median hemoglobin at diagnosis, g/L (range)	76 (53–142)	74 (32–147)	0.361
Median platelets at diagnosis, ×10^9^/L (range)	59 (30–134)	34 (4–447)	0.016
FAB, no.			0.060
M1	1	21	
M2	4	73	
M3	0	24	
M4	2	28	
M5	5	17	
M6	0	7	
Karyotype classification			0.001
Favorable	0	51	
Intermediate	12	86	
Poor	0	19	
No data	0	14	
MDS	4	47	
Sex, male/female	3/1	26/21	0.625
Median age at diagnosis, years (range)	70 (59–76)	52 (15–84)	0.107
Median WBC at diagnosis, ×10^9^/L (range)	2.8 (2.1–8.9)	3.1 (1.2–37.9)	0.986
Median hemoglobin at diagnosis, g/L (range)	64 (57–118)	63 (26–130)	0.547
Median platelets at diagnosis, ×10^9^/L (range)	58 (36–119)	66 (10–1176)	0.808
WHO, no.			0.446
5q-	0	4	
RA/RARS	0	7	
RCMD/RCMD-RS	1	20	
RAEB-1	1	8	
RAEB-2	2	8	
Karyotype classification			0.481
Favorable	4	34	
Intermidiate	0	7	
Poor	0	4	
No data	0	2	
IPSS			0.206
Low	0	6	
Int-1	2	31	
Int-2	2	5	
High	0	5	

WBC indicates white blood cell count at diagnosis; IPSS, International Prognostic Scoring System; WHO, World Health Organization; FAB, French-American-British classification; RA, refractory anemia; RARS, refractory anemia with ringed sideroblasts; RCMD, refractory cytopenia with multilineage dysplasia; RCMD-RS, refractory cytopenia with multilineage dysplasia with ringed sideroblasts; RAEB, refractory anemia with excess of blasts;

**Table 2:** The clinical and hematopoietic parameters of 16 patients with *DNMT3A* R882 mutations.

ID	Sex/Age (years)	Diagnosis	WBC(×10^9^/L)	Haemoglobin(g/L)	Platelet(×10^9^/L)	Karyotype	*DNMT3A* mutation
1	M/49	AML-M2	2.7	59	47	N	R882H
2	F/57	AML-M5	76.1	73	49	N	R882C
3	M/60	AML-M2	1.3	60	65	N	R882H
4	M/56	AML-M5	154.1	78	35	N	R882H
5	F/70	AML-M5	135.4	65	77	N	R882H
6	M/93	AML-M5	197.7	110	69	−17	R882C
7	M/67	AML-M4	107.0	53	118	N	R882H
8	M/63	AML-M1	88.3	104	30	+21	R882P
9	M/61	AML-M4	37.7	105	119	N	R882H
10	M/54	AML-M2	10.7	90	40	N	R882H
11	M/70	AML-M5	1.6	142	79	N	R882H
12	M/54	AML-M2	73.4	68	134	N	R882C
13	F/76	RAEB-2	2.8	65	47	N	R882H
14	M/59	RCMD	8.9	62	70	N	R882H
15	M/63	RAEB-2	2.7	118	119	N	R882C
16	M/76	RAEB-1	2.1	57	36	N	R882H

Furthermore, *DNMT3A* R882 mutations were found more frequently among monoblastic leukemia (M4 and M5, 7 of 52, 13.4%) compared to non-monoblastic leukemia (M1, M2, M3 and M6, 5 of 130, 3.8%) (*P* = 0.041). This result further confirmed the specificity of *DNMT3A* R882 mutations in monocytic lineage [3], [9].

In a total of 168 patients with cytogenetic data, all 12 *DNMT3A* R882 mutations were observed in patients with intermediate-risk karyotype (Table 1), mainly in patients with normal karyotypes (10 of 12 mutations, 83.3%). Two cytogenetically abnormal patients harbored monosomy 17 and trisomy 21, respectively. Among the patients with normal karyotypes, 13.2% (10 of 76) cases showed R882 mutation, significantly higher than 2.2% (2 of 92) in those with chromosomal abnormalities (*P* = 0.007). No R882 mutation was seen in patients with t(8;21), t(15;17), and other recurrent chromosomal abnormalities, such as translocations involving chromosome 11q23 and deletions involving chromosome 5 or 7.

**Figure 5:**
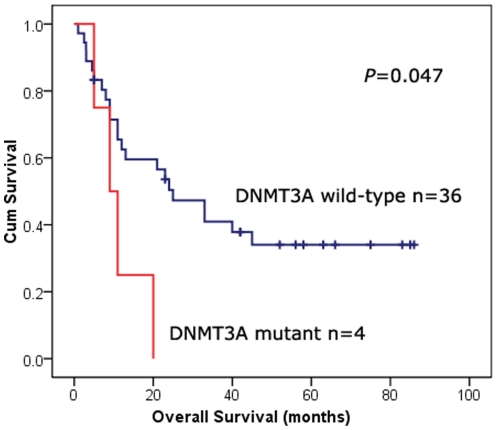
Overall survival of MDS patients divided according to *DNMT3A* mutation status at diagnosis.

4 (7.8%) heterozygous *DNMT3A* R882 mutations were also identified in MDS, including 3 R882H and 1 R882C mutations. The difference of age and hematologic parameters was not seen between patients with and without mutations. However, all 4 patients were identified with normal karyotype and were classified in intermediate-risk group according to IPSS classification. To investigate the prognostic impact of *DNMT3A* mutations in MDS, 40 cases with follow-up data were considered for survival analysis. The median follow-up of these patients was 23 months. The overall survival (OS) of MDS patients with *DNMT3A* mutation (median 9 months, 95% confidence interval 3–15 months) was shorter than those without mutation (median 25 months, 95% confidence interval 12–38 months) (*P* = 0.047, Figure 5). A recent study also identified 13 *DNMT3A* mutations in 12/150 (8.0%) MDS patients [4]. R882 mutations were found in only 4 cases. Correlation was not found between *DNMT3A* mutations and FAB subtypes, karyotypes, or IPSS subgroups, suggesting that *DNMT3A* mutation is an early genetic event in MDS. However, *DNMT3A* mutations were shown to contribute to a worse overall survival [4]. An association of *DNMT3A* mutations with poor prognosis was also observed in AML-M5 patients [9].

It should be noted that *DNMT3A* mutations in other nucleotide sites were not found in our AML and MDS patients. Two groups discovered a high frequency of non-R882 mutations in AML and MDS (40.3% and 69.2%, respecitively) [3], [4]. However, Yan et al observed only 13.0% (3/23) non-R882 mutations in Chinese AML-M5 patients [9]. No *DNMT3A* mutations were detected in pediatric AML by Ho et al [10]. These studies indicate that the *DNMT3A* mutagenesis may differ within different races and be associated with patient's age.

Three recent studies have identified *DNMT3A* mutations in 6.7% PV, 4.4%–15% MF (including PMF and post-ET/PV MF) and 0%–3.8% CMML [11]–[13]. No *DNMT3A* mutations were found in all of our MPN and CMML patients. However, the sample size was so small, more cases should be studied to determine the exact frequency of *DNMT3A* mutations in Chinese MPNs and CMML.

The catalytic activity relies on the methyltransferase (MTase) domain of DNMT3A protein, which contains ten blocks of conserved amino acid motifs [14], [15]. The R882 residue, located in front of motif X [15], [16], is considered to participate in the homodimerization and activation of the protein [17]. Mutations at R882 residue inhibit both DNA binding and catalytic activity [8], [14]. Overexpression of *DNMT3A* has been reported in various malignancies including AML [2]. Two recent studies have revealed that depletion of *DNMT3A* inhibits tumor cell proliferation and metastasis [17], [18]. Moreover, DNMT inhibitors such as 5-aza-2′-deoxycytidine induce cancer cell apoptosis through the covalent binding to DNMT3A and DNMT3B [19]. All of these findings suggest that *DNMT3A* functions as an oncogene. It seems that R882 mutations act in a dominant-negative fashion to reduce the MTase activity of the enzyme. However, the absence of global changes in DNA methylation in *DNMT3A*-mutated AML indicates that there are other molecular mechanisms contributing to leukemogenesis besides abnormal DNA methylation. More works are required to determine the functional consequences of R882 mutations and the underlying mechanisms.
